# Biosynthesis of Fatty Alcohols in Engineered Microbial Cell Factories: Advances and Limitations

**DOI:** 10.3389/fbioe.2020.610936

**Published:** 2020-12-03

**Authors:** Anagha Krishnan, Bonnie A. McNeil, David T. Stuart

**Affiliations:** Department of Biochemistry, University of Alberta, Edmonton, AB, Canada

**Keywords:** fatty alcohol, metabolic engineering, fatty alcohol reductase, carboxylic acid reductase, *E. coli*, *S. cerevisiae*, yeast, cyanobacteria

## Abstract

Concerns about climate change and environmental destruction have led to interest in technologies that can replace fossil fuels and petrochemicals with compounds derived from sustainable sources that have lower environmental impact. Fatty alcohols produced by chemical synthesis from ethylene or by chemical conversion of plant oils have a large range of industrial applications. These chemicals can be synthesized through biological routes but their free forms are produced in trace amounts naturally. This review focuses on how genetic engineering of endogenous fatty acid metabolism and heterologous expression of fatty alcohol producing enzymes have come together resulting in the current state of the field for production of fatty alcohols by microbial cell factories. We provide an overview of endogenous fatty acid synthesis, enzymatic methods of conversion to fatty alcohols and review the research to date on microbial fatty alcohol production. The primary focus is on work performed in the model microorganisms, *Escherichia coli* and *Saccharomyces cerevisiae* but advances made with cyanobacteria and oleaginous yeasts are also considered. The limitations to production of fatty alcohols by microbial cell factories are detailed along with consideration to potential research directions that may aid in achieving viable commercial scale production of fatty alcohols from renewable feedstock.

## Introduction

The use of microbial cells for the production of oleochemicals, particularly of single cell oil (SCO), has been of interest since the second half of the twentieth century as an alternative to traditional petrochemical production. With the rise of synthetic biology and metabolic engineering, particularly in the last decade, focus has shifted to the overproduction of specialized oleochemicals by engineered microbial cell factories. Use of microbial cultivations from renewable feedstock for the production of oleochemicals has the potential to address many of the environmental and supply chain concerns that are increasingly associated with traditional petrochemical and oil crop production. However, relatively low production yields and high costs associated with feedstock, cultivation and extraction, hinder progress of these technologies toward commercialization. This is largely due to the fact that many oleochemicals sell for relatively low commodity prices, which can be comparable to those of the purified sugar feedstock used, and that current yields fall below the maximum theoretical yield for production. This is exacerbated by the fact that lipid production is in most cases decoupled from cell growth, meaning that a substantial portion of carbon derived from the feedstock is channeled into biomass accumulation rather than the desired lipid product synthesis. Therefore, strategies for increasing yields, decreasing costs of cultivation using low-cost feedstock and simplifying product recoveries are among the measures that will be necessary to generate a commercially viable microbial oleochemical production process at current commodity prices.

Fatty alcohols are oleochemicals composed of hydrophobic acyl chains with hydroxyl moieties that provide them with amphiphilic properties (Hill et al., 1954). In nature, fatty alcohols are produced by a wide-variety of organisms. Fatty alcohol in the form of cetyl alcohol was first isolated from sperm whale oil in 1817 by Chevreul (1823). In marine bacteria and algae they serve as energy reserves and provide buoyancy (Sargent and Gatten, 1976; Rontani et al., 1999). Terrestrial plants and insects also produce fatty alcohols as a component of cutin, which prevents desiccation from surfaces, and birds and mammals produce them to form wax esters that are secreted to waterproof feathers and fur (Jacob, 1976; Tulloch, 1976; Buckner et al., 1996). Fatty alcohols are also components of ether glycerophospholipids in mammals and act as signaling molecules for some insect species (Roelofs, 1995; Buckner et al., 1996; Cheng and Russell, 2004). Despite their widespread synthesis, fatty alcohols in their free form are relatively rare and found in low abundance in nature.

The amphiphilic nature of fatty alcohols makes them excellent chemical components of detergents, emulsifiers, and emollients in personal care products such as soaps, shampoos and creams. The current global market for fatty alcohol production is estimated at 6.8 billion USD and is expected to reach greater than 10 billion USD by 2023 (Global InfoResearch, 2020). Commercial production of fatty alcohols is predominantly achieved either through chemical conversion of fatty acids derived from oil crops, such a palm oil (natural fatty alcohols), or via synthesis from petrochemical feedstock (synthetic) (Hill et al., 1954). While these processes are efficient, they present environmental, social and economic challenges. Expansion of monoculture palm oil plantations drives loss of rain forests and biodiversity, while petrochemical synthesis is associated with increased carbon emissions and chemical waste (Wake, 2005; Fitzherbert et al., 2008; Lees and Vieira, 2013). Chemical conversion processes for the production of fatty alcohols have been extensively reviewed elsewhere (Munkajohnpong et al., 2020) and this review will focus exclusively upon efforts to engineer microbial cells to produce fatty alcohols.

## Microbial Metabolic Pathways Leading to Fatty Alcohol Production

Microbial production of fatty alcohols is dependent on both (i) the availability of substrates (fatty acyl-Coenzyme A (acyl-CoA), fatty acyl-ACP or free fatty acids and the redox donor, NADPH) and (ii) an enzyme to convert the substrate to the desired fatty alcohol. The availability of substrates is at least in part governed by the organism’s endogenous metabolic pathways but may be improved through genetic engineering; while the enzymatic conversion step in all production chassis utilized to date employs heterologous protein expression. The following sections will address the basic tenants of fatty acid biosynthesis and give an overview of the enzymes that catalyze these conversions, providing context for the subsequent sections on metabolic engineering.

### Fatty Acid Biosynthesis: An Overview

**FIGURE 1:**
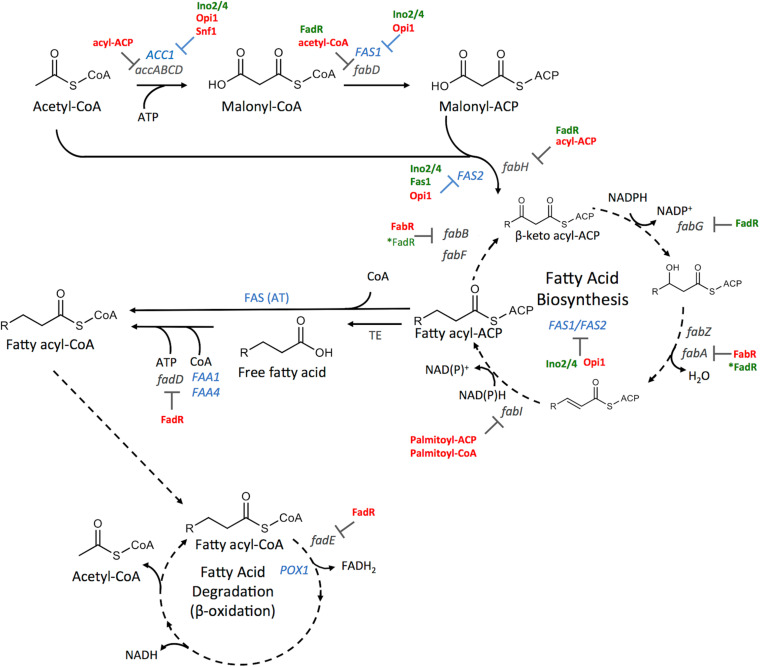
Overview of fatty acid biosynthesis (FAB) and degradation (FAD) highlighting enzymatic reactions and regulation steps from *E. coli* and *S. cerevisiae*. Briefly, acetyl-CoA carboxylase (*ACC1*, *accABCD*) converts acetyl-CoA to malonyl-CoA, which is then converted to malonyl-ACP (acyl carrier protein) and combined with acetyl-CoA by the activities of the fatty acid synthetase complex (*FAS1/2* and *fabA* (3-hydroxydecanoyl-acp dehydratase), *fabB* (3-oxoacyl-acp synthase 1), *fabD* (Malonyl CoA-acyl acp transacylase), *fabF* (3-oxoacyl-acp synthase 2), *fabG* (3-oxoacyl-acp reductase), *fabH* (3-oxoacyl-acp synthase 3), *fabI* (Enoyl-acp reductase), *fabZ* (3-hydroxyacyl-acp dehydratase)). Intrinsic acyl transferase (AT) activity of FAS converts fatty acyl-ACP to fatty acyl-CoA (Co-enzyme A) in *S. cerevisiae*, whereas, thioesterase (TE) activity is required in *E. coli* followed by activation by *fadD* (long-chain-fatty-acid-CoA ligase). Free fatty acids can be activated by Fatty Acid Activation proteins (*FAA1/4*) in *S. cerevisiae*. The first step of fatty acid degradation (FAD) is controlled by the gene production of *POX1* (peroxisomal fatty-acyl CoA oxidase) or *fadE* (Acyl-CoA dehydrogenase). Enzymes present in *S. cerevisiae* are shown in blue while the *E. coli* enzymes are shown in gray. Inhibitory regulation whether at the protein level or at the level of transcription is shown in red and activators are indicated in green. The transcriptional regulators Ino2/4 (INOsitol requiring transcription factors), Opi1 (OverProducer of Inositol) and Snf1 (Sucrose NonFermenting protein 1; an AMP-activated S/T protein kinase) are involved in *S. cerevisiae* and the Fatty acid metabolism regulator protein (FadR) and transcriptional repressor FabR are involved in *E. coli.*

While the general mechanism of fatty acid biosynthesis is conserved between prokaryotes and eukaryotes the molecular structure, organization, localization and compartmentalization of the enzymes participating in fatty acid synthesis may differ. Fatty acid biosynthesis in model organisms has been extensively reviewed (Tehlivets et al., 2007; Cronan and Thomas, 2009; Liu et al., 2011; Garay et al., 2014; Janssen and Steinbuchel, 2014; Beld et al., 2015; Fakas, 2017; Ruffing, 2017) and is only briefly described here. The primary enzymatic steps and regulatory points in fatty acid synthesis are illustrated in Figure 1.

#### Fatty Acid Biosynthesis and Degradation

*De novo* fatty acid biosynthesis begins with the carboxylation of acetyl-CoA to malonyl-CoA catalyzed by an ATP-dependent acetyl-CoA carboxylase (*ACC*) (Figure 1). In yeast, a single gene, *ACC1*, encodes the ACC activity, whereas in *E. coli* the ACC complex (accABCD) is encoded by four genes, *accA*, *accB*, *accC*, and *accD* (Janssen and Steinbuchel, 2014). With acetyl-CoA as the starter unit, and malonyl-CoA as the extender, the fatty acid synthase complex (FAS) catalyzes fatty acid synthesis using acyl carrier protein (Acp) as a tether (Figure 1). Yeasts use a cytosolic Type I FAS encoded by two genes, *FAS1* and *FAS2* whose protein products assemble into an α_6_β_6_ heterododecamer (Lomakin et al., 2007; Tehlivets et al., 2007). *E. coli* uses a Type II FAS complex composed of monofunctional enzymes encoded by nine separate genes (*fabA*, *fabB*, *fabD*, *fabF*, *fabG*, *fabH*, *fabI*, *fabZ*, *acp*) (Magnuson et al., 1993; Zhang and Rock, 2008; Janssen and Steinbuchel, 2014). Acetyl-CoA is generally the priming unit for FAS. However, propionyl-CoA or degradation products of branched chain amino acids can also be used as starter units resulting in odd or branched chain fatty acids respectively (Choi et al., 2000). Post-elongation, fatty acyl chains may either be (a) directly transferred from Acp to CoA by an intrinsic acyltransferase (AT) yielding long-chain acyl-CoA as in yeast, (b) directly used as a substrate for lipid biosynthesis in bacteria or (c) released as free fatty acids using a thioesterase (TE) as in mammals (Tehlivets et al., 2007). The length of the fatty acid chain is controlled largely by the enzymatic activity that releases the fatty acyl molecules from FAS, however, they may be further elongated by fatty acid elongases in yeast and FabF in *E. coli* (Garwin and Cronan, 1980; Toke and Martin, 1996; Oh et al., 1997; Tehlivets et al., 2007). Various desaturases introduce double bonds in the saturated fatty acyl derivatives with the Δ9-desaturase, Ole1, filling the role in *S. cerevisiae* and FabA and FabB playing key roles in *E. coli* (Martin et al., 2007; Janssen and Steinbuchel, 2014). Once generated, fatty acyl-ACP, fatty acyl-CoA or free fatty acids can act as substrates for fatty alcohol production.

Fatty acid degradation (FAD) takes place via the β-oxidation pathway generating acetyl-CoA as the terminal product. β-oxidation exclusively uses acyl-CoA thioesters and not acyl-ACP. In *E. coli*, this process occurs in the cytosol by the combined action of FadD and FadE (Janssen and Steinbuchel, 2014). In yeast, β-oxidation occurs exclusively in the peroxisomes, and the process relies on peroxisomal fatty acyl-CoA importers (Pxa1/Pxa2) and a cadre of peroxisomal enzymes [reviewed in van Roermund et al. (2003)].

#### Regulation

Fatty acid metabolism is tightly regulated in both prokaryotes and eukaryotes. Multilayer control, including transcriptional, translational and substrate-level regulation is involved in limiting the supply of substrates (Tehlivets et al., 2007; Janssen and Steinbuchel, 2014). In *E. coli*, several genes participating in fatty acid metabolism are controlled at the level of transcription through the activities of the transcription factors FadR and FabR (Janssen and Steinbuchel, 2014; Figure 1). FadR negatively regulates the expression of genes involved in β-oxidation while activating the transcription of fatty acid biosynthesis genes including *fabA*, *fabB* and the *fabHDG* operon. Long-chain fatty acyl-CoA can bind directly to FadR resulting in de-repression of β-oxidation genes and inhibition of genes involved in fatty acid biosynthesis (Black et al., 2000). On the other hand, FabR represses the transcription of *fabA* and *fabB* in the presence of unsaturated fatty acids. In addition, long-chain acyl-ACP acts as feedback inhibitor β-ketoacyl-ACP synthase III (FabH), enoyl-ACP reductase (FabI) and Acc (Magnuson et al., 1993; Heath and Rock, 1996; Davis and Cronan, 2001) thus limiting the flux through the fatty acid biosynthesis pathway.

In *S. cerevisiae*, lipid biosynthesis involves several cellular compartments, which increases the complexity of regulation. Most of the genes involved in phospholipid synthesis including *ACC1*, *FAS1* and *FAS2* contain a *cis*-acting UAS*_INO1_* (inositol-sensitive upstream activating sequence) regulatory sequence allowing either activation by the Ino2/Ino4 complex or repression by Opi1 (Chen et al., 2007; Tehlivets et al., 2007). The stoichiometry between Fas1 and Fas2 subunits is further maintained through a mechanism whereby Fas1 regulates transcription of *FAS2* (Wenz et al., 2001). Acc1 is also regulated at the protein level by phosphorylation of two serine residues (S^659^, S^1157^) by the AMP-dependent protein kinase, Snf1, and these phosphorylation events decrease Acc1 activity (Shi et al., 2014). Finally, Acc1, Fas1 and Fas2 are inhibited by long chain acyl-CoA (Faergeman and Knudsen, 1997).

### Fatty Alcohol Generating Enzymes

**FIGURE 2:**
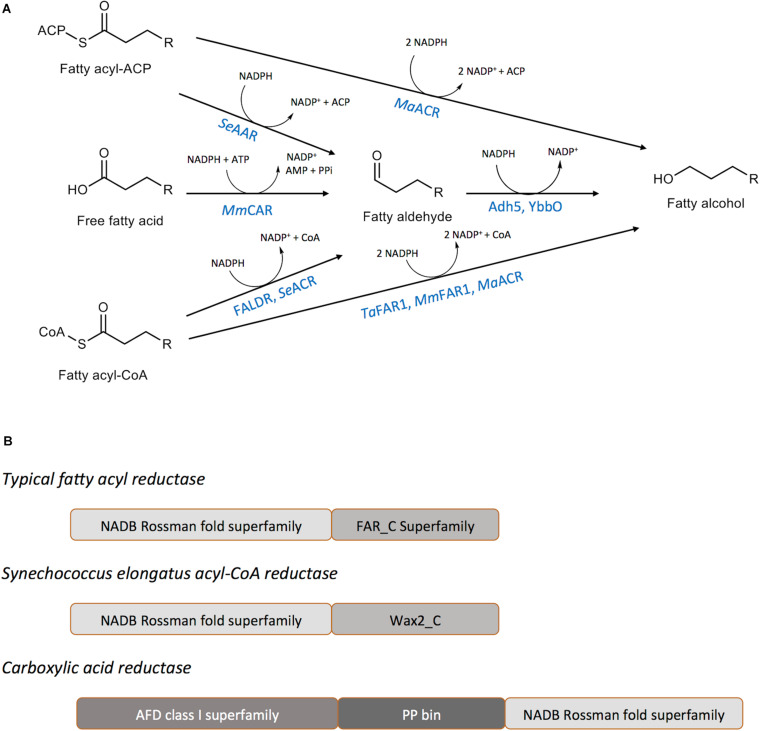
Enzymes catalyzing fatty alcohol formation. **(A)** Reaction schemes for fatty alcohol synthesis from common precursors indicating the enzymes required and intermediates generated. *MaFAR*, *Marinobacter aquaeolei* VT8 acyl-CoA reductase; Adh5, *S. cerevisiae* alcohol dehydrogenase 5; YbbO, *E. coli* aldehyde reductase; *MmCAR*, *Mycobacterium marinum* carboxylic acid reductase; FALDR, fatty aldehyde forming reductase; *TaFAR1*, *Tyto alba* fatty acyl-CoA reductase 1; *MmFAR1*, *Mus musculus* fatty acyl-CoA reductase 1. **(B)** Domains of the common enzymes utilized for the production of fatty alcohols. Typical FARs and *Synechococcus elongatus* acyl-CoA reductase enzymes include an amino-terminal NAD(P)H/NAD(P)+ binding (NADB) Rossman fold domain. Carboxyl-terminal domains belong to either the FAR-C or WAX2-C super families that are common among short-chain reductases. Carboxylic acid reductase enzymes possess a large amino-terminal adenylate forming Class I superfamily domain (AFD), a central phosphopantetheine binding site (PP bin), and a carboxyl-terminal large NADB Rossman fold.

Biological production of fatty alcohols can occur via the reduction of activated forms of fatty acyl thioesters (fatty acyl-CoA or fatty acyl-ACP) catalyzed by a fatty acyl reductase (FAR) (Reiser and Somerville, 1997; Cheng and Russell, 2004; Mudge et al., 2008; Steen et al., 2010; Hellenbrand et al., 2011; Rowland and Domergue, 2012) or by the reduction of free fatty acids, catalyzed by the enzyme carboxylic acid reductase (CAR) (Akhtar et al., 2013; Figure 2A). A third pathway, catalyzing the reduction of fatty acids to fatty aldehyde through an acyl-protein intermediate is known to occur in the luminescent bacterium *Photobacterium phosphoreum* (Riendeau et al., 1982; Rodriguez et al., 1983). The first two types of enzymes, FAR and CAR, have been extensively explored for the purposes of microbial fatty alcohol production (Fillet and Adrio, 2016) and will be our focus in this review.

#### Fatty Acyl Reductases (FAR)

Fatty acyl reductases (FARs) belong to a large family of reductases catalyzing the reduction of activated forms of fatty acids, preferentially using NADPH as the electron donor (Rowland and Domergue, 2012). The proteins share two distinct domains: (a) an NAD(P)H binding Rossmann-fold domain containing the active site motif, YXXXK and (b) a fatty acyl-CoA reductase domain (Rowland and Domergue, 2012; Figure 2B). Based on the terminal product, fatty acyl reductases can be divided into two classes: (i) fatty alcohol forming reductase (FAR) catalyzing the four-electron reduction of fatty acyl-CoA/ACP to fatty alcohols and (ii) fatty aldehyde forming reductases (FALDR; consisting either of acyl-CoA reductases (ACR) or acyl-ACP reductases (AAR)) catalyzing a two-electron reduction to fatty aldehydes which can be further reduced to fatty alcohols by the action of endogenous aldehyde reductases (AHR) or alcohol dehydrogenases (ADH). Either fatty acyl-CoA or fatty acyl-ACP can serve as substrates for FARs (Table 1). Of special note, the *Arabidopsis thaliana* Far2, Far6 and *Marinobacter aquaeolei* VT8 FAR (referred to here as *MaFAR* but referred to throughout the literature as either MaFAR2, Maqu_2220, FaCoAR, *MhFAR* or *MaACR*) are bifunctional enzymes capable of utilizing both fatty acyl-CoA and fatty acyl-ACP as substrates (Hofvander et al., 2011; Willis et al., 2011; Doan et al., 2016). Most FARs characterized, show a preference for long-chain (> C12) to very-long-chain acyl-CoA substrates (Table 1; Wang and Kolattukudy, 1995; Reiser and Somerville, 1997; Vioque and Kolattukudy, 1997; Rowland et al., 2006; Doan et al., 2009; Domergue et al., 2010; Hofvander et al., 2011; Willis et al., 2011; Teerawanichpan and Qiu, 2012). However, *MaFAR* is less selective and can act on a wide range of fatty acyl chain lengths (Hofvander et al., 2011). Additionally, both membrane-associated and soluble FARs have been identified and characterized (Wang and Kolattukudy, 1995; Reiser and Somerville, 1997; Vioque and Kolattukudy, 1997; Metz et al., 2000; Willis et al., 2011; Lin et al., 2013).

**TABLE 1:** Fatty alcohol generating enzymes.

**Organism**	**Enzyme**	**Carbon chain length (Catalytic efficiency)#**	**Electron donor**	**Substrate**	**Product**	**References**
*Acinetobacter calcoaceticus*	ACR1, fatty acyl reductase	C16:0 > C18:0 > C14:0 > C20:0 > C22:0	NADPH	Fatty acyl-CoA	Fatty aldehyde	Reiser and Somerville, 1997
*Synechococcus elongatus*	ACR, fatty acyl reductase	C18:0 > C20:0 > C18:1 = C16:0 > C12:0	NADPH	Fatty Acyl-CoA	Fatty aldehyde	Lin et al., 2013
*Synechococcus sp. PCC 7002*	AAR, fatty acyl-ACP reductase	Only C18:0 measured	NADPH	Fatty acyl ACP >> fatty-Acyl CoA	Fatty aldehyde	Steen et al., 2010
Honey bee *Apis mellifera*	FAR, fatty acyl CoA reductase	C18:0 > C22:0 > C20:0	NADPH	Fatty acyl-CoA	Fatty alcohol	Teerawanichpan and Qiu, 2010
Mouse *Mus musculus*	FAR, fatty acyl CoA reductase	C16:0, C18:0, C18:1, C18:2	NADPH	Fatty acyl CoA	Fatty alcohol	Cheng and Russell, 2004
Human *Homo Sapien*	FAR, fatty acyl CoA reductase	C16:0, C18:0, C18:1, C18:2	NADPH	Fatty acyl CoA	Fatty alcohol	Cheng and Russell, 2004
Barn owl *Tyto alba*	FAR1, fatty acyl CoA reductase	C16:0 >> C18:0	NADPH	Fatty acyl-CoA	Fatty alcohol	Hellenbrand et al., 2011
*Arabidopsis thaliana*	CER4, fatty acyl CoA reductase	* C24:0, C26:0	NADPH	Fatty acyl-CoA	Fatty aldehyde, Fatty alcohol	Rowland et al., 2006
*A. thaliana*	DPW, fatty acyl-ACP reductase	C16:0	NADPH	Fatty acyl-ACP >> fatty acyl-CoA	Fatty alcohol	Shi et al., 2011
Jojoba *Simmondsia chinensis*	FAR, fatty acyl CoA reductase	C16:0	NADPH	Fatty acyl-CoA	Fatty alcohol	Metz et al., 2000
*A. thaliana*	MS2, fatty acyl ACP reductase	C16:0 >> C18:0	NADPH, NADH (low efficiency)	Fatty acyl-ACP	Fatty alcohol	Chen et al., 2011
*A. thaliana* chloroplast localized	FAR, fatty acyl CoA reductase	C16:0	NADPH	Fatty acyl-CoA >> Fatty acyl-ACP	Fatty alcohol	Doan et al., 2016
*Marinobacter aquaeolei VT8*	*Maqu_2507*, fatty acyl-reductase	C16:0 > C16:1 > C20:4 > C18:1 > C18:0 > C14:0 > C12:0 > C8:0	NADPH	Fatty acyl CoA/ACP	Fatty alcohol	Willis et al., 2011
*M. aquaeolei VT8*	*MaFAR*, fatty acyl-reductase	C18:1 > C20:0 > C18:0 > C16:0 > C16:0-ACP = ^†^Ric-CoA = C22:1	NADPH	Fatty Acyl CoA/ACP	Fatty alcohol	Hofvander et al., 2011
*Mycobacterium marinum*	CAR carboxylic acid reductase	C12:0 > C10:0 > C10:0 > C8:0 > C6:0 >> C4:0	NADPH	Fatty acid	Fatty aldehyde	Akhtar et al., 2013

*Measured *in vivo* by expressing the gene in yeast. ^†^Ricinoleoyl-CoA. ^#^Determined based on *in vivo* or *in vitro* assays.

#### Carboxylic Acid Reductases (CARs)

CARs have a broad substrate scope and have been employed for production of numerous industrially valuable aldehydes and alcohols (Butler and Kunjapur, 2020). They are relatively large, multi-domain enzymes possessing an amino-terminal adenylation domain, a carboxyl-terminal thioester reductase domain, and a central phosphopantetheine-binding domain (Figure 2B; Finnigan et al., 2017). CARs catalyze the reduction of a carboxylic acid to an aldehyde at the expense of adenosine triphosphate (ATP) and NADPH to produce adenosine monophosphate (AMP), pyrophosphate (PPi), and NADP^+^ as by-products (Akhtar et al., 2013; Finnigan et al., 2017). The hydrolysis of ATP makes the reduction of acids to aldehydes by CARs strongly thermodynamically favorable. *Mycobacterium marinum* CAR (hereafter *MmCAR*) has been effectively coupled to an alcohol dehydrogenase, to provide a complete reduction of acyl-CoA to the acyl-alcohol (Akhtar et al., 2013). Although *MmCAR* is active against a wide range of substrates it also requires phosphopantetheinyl transferase, often provided by *B. subtilis Sfp*, for full activity (Venkitasubramanian et al., 2007).

## Metabolic Engineering of Microbial Hosts for Fatty Alcohol Production

Progress in metabolic engineering, synthetic, and systems biology have allowed the rewiring of microbial metabolism to produce fatty alcohols possessing various desired physicochemical characteristics. With the flexibility associated with metabolic pathway engineering and a relatively low environmental footprint when compared to the existing petrochemical or oleochemical production counterparts, microbial synthesis is becoming an attractive alternate production approach (Zhou et al., 2014). Engineering of microbes for the production of fatty acid-based products have been extensively reviewed (Pfleger et al., 2015; Cho et al., 2020; Yan and Pfleger, 2020). Of these, FFA production has been most successful with yields of up to 70–80% theoretical limit achieved in *E. coli* (Dellomonaco et al., 2011; Zhang et al., 2012). Production of fatty alcohols and fatty acids both depend on the synthesis of acyl chains and thus the successful metabolic engineering strategies for FFA production can serve as a potential starting point for fatty alcohol synthesis. However, as many enzymes utilized in production of fatty alcohols use acyl-ACP or acyl-CoA as substrates rather than FFA, it must be kept in mind that production of FFA can also act as a competitive carbon sink for production of fatty alcohols.

### Engineering Fatty Alcohol Production in *Escherichia coli* and Cyanobacteria

*E. coli* has been a prominent host for production of a range of biochemicals including fatty alcohols (Wang et al., 2017). *E. coli* does not produce fatty alcohols naturally; however, heterologous enzyme expression has resulted in fatty alcohol producing strains. As a production chassis, *E. coli* has several advantages over other industrial microbes including high growth rates, ability to grow in minimal media using a variety of carbon sources, capacity for aerobic and anaerobic growth, rapid genetic manipulation and availability of genetic tools and databases (Wang et al., 2017). In addition, photosynthetic microbes, particularly cyanobacteria, have attracted interest as cell factories for their ability to capture light energy and fix CO_2_ for conversion into a variety of biochemicals. Like *E. coli*, cyanobacteria are prokaryotic, lack organelles and do not accumulate neutral lipids; however, they possess robust lipid biosynthetic metabolism necessary for building their thylakoid membranes (Liu et al., 2011). While the trophic modes are highly different in the two organisms, their fatty acid metabolism is similar. Most of the metabolic engineering targets used in *E. coli* can and have been successfully applied for boosting cyanobacterial fatty alcohol production.

#### Enzyme Selection

Heterologous enzyme activity is a common bottleneck in the engineering of metabolic pathways (Peralta-Yahya et al., 2012). Therefore, testing multiple enzymes for activity in the host chassis is critical to optimize production. Reductases from soil, marine and photosynthetic bacteria (Reiser and Somerville, 1997; Steen et al., 2010; Hofvander et al., 2011; Willis et al., 2011; Akhtar et al., 2013; Lin et al., 2013), as well as plants (Doan et al., 2009; Shi et al., 2011; Rowland and Domergue, 2012) have been tested in *E. coli* for the production of fatty alcohols (Table 2). In *E. coli*, the *M. aquaeolei* VT8 *MaFAR* enzyme and related *Maqu_2507* that catalyze the 4 electron conversion of acyl-ACP or acyl-CoA to acyl-alcohols were found to outperform the *Acinetobacter sp. M-1* acyl-CoA reductase Acr1 that catalyzes the 2 electron conversion to acyl-aldehydes (Liu et al., 2013). However, the expression of soluble acyl-aldehyde producing Aar1 from *Synechococcus elongatus* in conjunction with the endogenous aldehyde reductase YbbO has yielded titers of up to 1.98 g L^–1^ in fed batch cultures (Fatma et al., 2016, 2018). On the other hand, the carboxylic acid reductase *Mm*Car has been reported to produce 350 mg L^–1^ fatty alcohols (Akhtar et al., 2013). As Aar does not consume ATP or require thioesterase activity for aldehyde production it may provide an energetic advantage when carbon flux through the acyl-ACP biosynthetic pathway is maximized (Nozzi et al., 2014; Liu et al., 2016).

**TABLE 2:** Fatty alcohol production in *E. coli.*

**Properties**	**Host strain**	**Product-forming enzyme**	**Mutations**	**Media**	**Shake flask titer (mg L^–1^)**	**Fed batch titer (mg L^–1^)**	**Productivity (mg L^–1^h^–1^)**	**Yield (g/g)**	**References**
Even chain C12–C18	C41 (DE3)	*AcACR1*	*‘tesA*, *fadD*, *ΔfadE*	M9	60	ND	ND	ND	Steen et al., 2010
	BL21 (DE3)	*AbACR1*	*BTE*, *fadD*	M9	37.5	598	24.94	0.001	Zheng et al., 2012
	MG1655	*MaFAR*	*3X BTE*, *FadD*	M9	ND	1650	13.75	0.134	Youngquist et al., 2013
	BL21(DE3)	*MaFAR*	*‘tesA*, *fadD*	M9	422	1750	16	0.028	Liu et al., 2013
	BL21 (DE3)	*MmCAR*	*‘tesA*, *AHR*, *Bssfp*	Minimal	350	ND	ND	0.05	Akhtar et al., 2013
	MG1655	*MaFAR*	*fadD*, ‘*tesA*, *ΔfadE*, *araD*:*acpS* (*C. glutamicum*)	M9	3500	ND	50	0.126	Haushalter et al., 2015
	DH5α	*SeAAR*	*ybbO*, *fadR*, *ΔplsX*, *fabZ*	M9	270	1989	41.43	ND	Fatma et al., 2016
	MG1655	*MaFAR*	*ΔtesCB*, *ΔldhA*, *Δpta*, *ΔackA*	LB	760	6330	126	ND	Liu et al., 2016
	MG1655	*MaFAR*	*ΔfadE*	M9 (2mM NH_4_^+^)	180	ND	ND	0.12	Chubukov et al., 2017
	DH5α	*SeAAR*	*zwf*, *ybbO*, *Δedd*, *Δpps*, *ΔldhA*, *ΔaceA*, *ΔpoxB*, *Δpta*, *ΔpflB*, *ΔplsX*	Modified mineral medium	1506	12500	ND	ND	Fatma et al., 2018
Octanol	MG1655	*MaFAR*	*ΔaraBAD*, *ΔfadE*, *ΔfadD::*P_TRC_CpFatB1*-*lacI*, *ΔackA-pta::P*_TRC_*-Mt-FadD6–lacI*, *ΔpoxB::P*_TRC_*-Mt-FadD6*, *ΔFadBA::P*_TRC_*Ma-ACR-lacI*	Mineral medium 2% glycerol	1273		53	0.064	Hernandez Lozada et al., 2020
Octanol	BL21 (DE3)	*Mm CAR*	*BsSFP*, *At tes3 AHR*	LB	62		4.4	12	Akhtar et al., 2015
Odd (C_2n–1_) chain	BL21 (DE3)	*O. sativa αDOX*	*‘tesA FadR*, *AHR*	M9 yeast extract, glycerol	105	1950	105	0.018	Cao et al., 2015
Branched chain	UB1005	*MaFAR*	Δ*fabH*, Δ*fadE*, Δ*ldhA*, *fadR*, *ilvCD*, *S. aureus fabH*, *B. subtilis lplA*, *leu* operon, and *bkd* operon	M9	170	350 branched chain, 737 total fatty alcohol	9	ND	Jiang et al., 2017

FAR enzymes from several sources have also been introduced into cyanobacteria to achieve fatty alcohol biosynthesis. In the cyanobacterium *Synechocystis* sp. PCC6803, heterologous expression of FAR genes from *Jojoba* and *Arabidopsis* generated 9.73 ± 2.73 μg OD^–1^ L^–1^ fatty alcohol (Tan et al., 2011). Integration of the bifunctional *MaFAR* into the *slr0168* locus of wild-type *Synechocystis* sp. PCC 6803 was found to produce 0.39 ± 0.06 mg g^–1^ DCW (Yao et al., 2014). These heterologous enzymes displayed activity but attempts to optimize expression of the FAR in cyanobacteria has not been reported.

It is not yet possible to define the most effective enzyme(s) for microbial production of fatty alcohols as the preferred substrates for each of these enzymes differ and reported studies have used a variety of different strain backgrounds, culture conditions and feedstock (Figure 2 and Table 2). However, owing to differences in substrate preference the subsequent metabolic engineering strategies that accompany the use of specific enzymes will be tailored differently.

#### Modulating Metabolism: Enhancing Precursor Availability and Eliminating Competition

**FIGURE 3:**
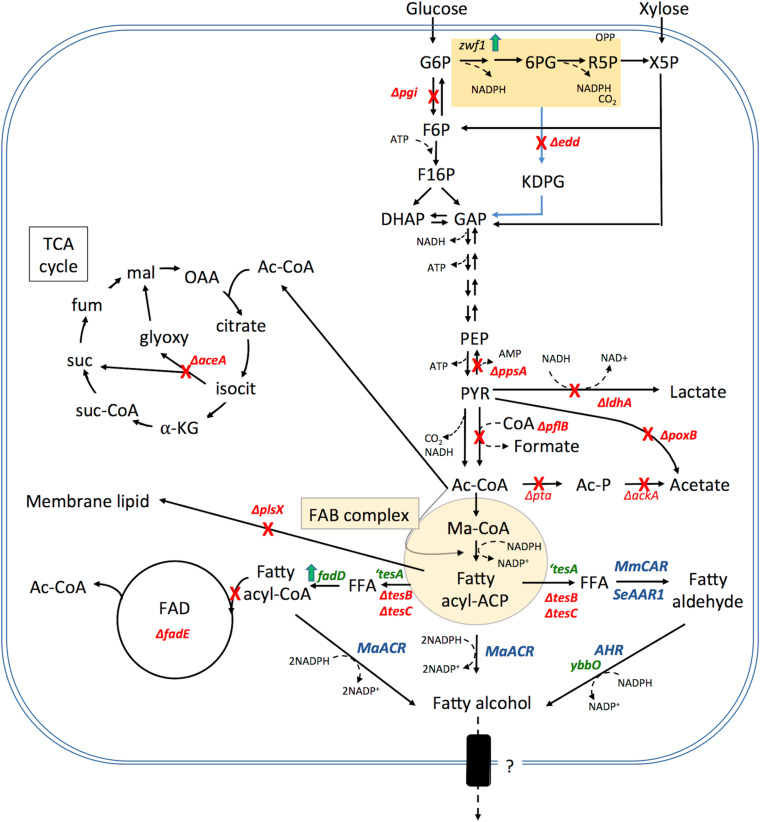
Summary of metabolic engineering performed for fatty alcohol production in *E. coli*. Genes that have been effectively inactivated (shown in red), overexpressed (shown in green) and heterologous enzyme expression (shown in blue) that have resulted in increased fatty alcohol production in *E. coli*. Cofactors are noted in particularly salient positions but are omitted from others for clarity. Note: these genetic modifications have not necessarily been combined into a single production strain. Abbreviation and gene names used are: G6P, glucose-6-phosphate; 6PG, 6-phosphogluconate; R5P, ribulose-5-phosphate; X5P, Xylulose-5-phosphate; OPP, oxidative pentose phosphate pathway; F6P, fructose-6-phosphate; F16P, fructose-1,6-bisphosphate; DHAP, dihydroxyacetone phosphate; GAP, glyceraldehyde-3-phosphate; PEP, phosphoenolpyruvate; PYR, pyruvate; Ac-CoA, acetyl Co-enzyme A; Ma-CoA, Malonyl CoA; KDPG, 2-Keto-3-deoxy-6-phosphogluconate; OAA, oxaloacetate; isocit, isocitrate; αKG, α-ketoglutarate; suc-CoA, succinyl-CoA; suc, succinate; fum, fumarate; mal, malate; glyoxy, glyoxylate; FFA, free fatty acid; Ac-P, acetyl phosphate; ACP, acyl carrier protein; FAB, fatty acid biosynthesis; FAD, fatty acid degradation; *zwf1*, Glucose-6-phosphate dehydrogenase; *pgi*, phosphoglucose isomerase; *edd*, phosphogluconate dehydratase; *ppsA*, phosphoenolpyruvate synthetase; *ldhA*, lactate dehydrogenase; *pflB*, pyruvate formate lyase; *poxB*, pyruvate oxidase; *ackA*, acetate kinase; *pta*, phosphate acetyltransferase; *tes*, thioesterase; *plsX*, phosphate acetyltransferase involved in phospholipid metabolism; *aceA*, isocitrate lyase; *ybbO*, NADP^+^-dependent aldehyde reductase; *AHR*, aldehyde reductase; *MmCAR*, *Mycobacterium marinum* carboxylic acid reductase; *SeAAR*, *Synechococcus elongatus* acyl-CoA reductase; *MaFAR*, *Marinobacter aquaeolei* VT8 acyl-CoA reductase; *fadD*, long-chain-fatty-acid-CoA ligase; *fadE*, Acyl-CoA dehydrogenase.

Most rational metabolic engineering efforts have been directed toward improving substrate availability and eliminating competing pathways. In *E. coli* expression of a heterologous FAR has consistently been engineered in conjunction with the deletion of the first step of β-oxidation (*ΔfadE*), expression of a cytoplasmic thioesterase and the overexpression of fatty acid-CoA ligase (*fadD*) (Figure 3). Expression of a cytoplasmic thioesterase, results in the hydrolysis of fatty acyl-ACP and production of free fatty acids (FFA). When combined with the overexpression of *fadD*, catalyzing the activation of FFA into fatty acyl-CoA, this results in an enhanced pool of available fatty acyl-CoA. Deletion of fatty acyl-CoA dehydrogenase/enoyl-CoA reductase (*fadE*), involved in the bacterial β-oxidation cycle, eliminates a fatty acyl-CoA competing pathway and increases available substrate for the FAR or CAR enzyme. The thioesterase most widely employed in *E. coli* is a modified version of the endogenous periplasmic thioesterase (*tesA*) lacking a leader sequence so that it localizes to the cytoplasm (‘*tesA*). Overexpression of ‘*tesA* also serves to lower the cellular acyl-ACP concentrations and the corresponding feedback inhibition to allow for increased flux through acyl-ACP/CoA synthesis (Cho and Cronan, 1995). Although overexpression of *‘tesA* and *fadD* increase the energetic cost of fatty alcohol production from acyl-ACP, they none-the-less substantially increase titers presumably by increasing flux through the acyl-ACP synthesis pathway (Fatma et al., 2018). Pioneering work by Steen et al. (2010) combined overexpression of *‘tesA*, *fadD* and the fatty aldehyde reductase (*acr1*) from *A. calcoaceticus*, in a Δ*fadE* strain resulting in 60 mg L^–1^ fatty alcohol. Replacing *acr1* with *MaFAR*, led to a strain producing 170–422 mg L^–1^ fatty alcohols under shake flask conditions and 1.725 g L^–1^ in fed batch culture (Liu et al., 2013). By optimizing the expression level of each of the above genes, Haushalter et al. achieved an eightfold higher production in shake flasks, 3.5 g L^–1^ with yields of up to 0.13 g/g glucose consumed (Haushalter et al., 2015). Similarly, in cyanobacteria the overexpression of the endogenous fatty acid-CoA ligase (*slr1609* gene or cyanobacterial *fadD*) enhanced fatty alcohol production by 60% (Gao et al., 2012).

To improve the fatty acyl-ACP pool available to *MaFAR* in *E. coli*, Liu et al. deleted the endogenous thioesterases, *tesC* and *tesB*, eliminating the conversion of fatty acyl-ACP to free fatty acids (Liu et al., 2016). The resulting strain displayed 70% higher fatty alcohol titer than the *MaFAR-*only expressing strain. In *Synechocystis* sp. PCC 6803, increasing fatty acyl-ACP availability by deletion of both the endogenous acyl-ACP reductase (*sll0209*) and aldehyde deformylating oxygenase (*sll0208*) increased fatty alcohol yield to 2.8 mg g^–1^ DCW (Yao et al., 2014).

In *E. coli*, knocking out competing carbon sinks including lactate and acetate production through the deletion of lactate dehydrogenase (*ldhA*), phosphate acetyltransferase (*pta*) and acetate kinase (*ackA*) increased fatty alcohol titer by 162% resulting in 760 mg L^–1^ in shake flask and 6.33 g L^–1^ in a fed-batch fermentation (Liu et al., 2016). Fatma et al. (2018), have used a flux balance constraint-based modeling and iterative simulation approach to identify the gene targets that have the greatest impact on fatty alcohol production (Fatma et al., 2018). Enhancing the flux through the pentose phosphate pathway by deletion of the Entner-Duodoroff pathway gene phosphogluconate dehydratase (*Δedd*) and the overexpression of *zwf1* (glucose-6-phosphate dehydrogenase) allowed for improved reductant availability. Further deletion of alternate carbon sinks including acetate (Δ*poxB*, Δ*pta*), formate (Δ*pflB*), lactate (Δ*ldhA*), anaplerotic PEP pathway (gluconeogenesis) (Δ*pps*, phosphoenolpyruvate synthase) and glyoxylate shunt (Δ*aceA*) were sequentially combined to improve fatty alcohol production. Finally, deletion of *plsX* (involved in phospholipid metabolism) in the above strain resulted in a 1.95-fold increase in fatty alcohol production in shake-flasks giving a titer of 1.5 g L^–1^. However, stacking of Δ*plsX* slowed growth and limited biomass accumulation. Scale-up of fatty alcohol production from this strain in a controlled fed-batch cultivation resulted in the final titer of 12.5 g L^–1^ fatty alcohol.

Similarly, in cyanobacteria, blocking competitive carbon sinks has also proven to be an effective strategy to increase fatty alcohol production. Eliminating glycogen (Δ*agp*) and poly-β-hydroxybutyrate (Δ*phaAB*) synthesis along with multicopy integration of the *Jojoba* and *A. thaliana* FAR resulted in a fatty alcohol yield of 761 ± 216 μg g^–1^ dry cell weight (DCW) (Qi et al., 2013). Recently, CRISPRi-mediated repression of the genes involved in acyl-ACP consuming pathways including hydrocarbon biosynthesis was combined with heterologous expression of *MaFAR* leading to a fatty alcohol production of 10.3 mg g^–1^ DCW (Kaczmarzyk et al., 2018). Particularly, a 90% repression of the essential acyl-transferase *PlsX* led to a threefold increase in the fatty alcohols produced.

Fatty alcohol synthesis using CAR has been pursued in *E. coli* originally by Akhtar et al. (2013). In this system, four gene products were expressed: (a) *‘tesA*, for metabolic overaccumulation of free fatty acids, (b) *Bacillus subtilis Sfp* for activation of Car, (c) *M. marinum Car* for activation and subsequent reduction of the free fatty acids to fatty aldehyde and (d) *Ahr* from *E. coli* for reduction of fatty aldehyde to fatty alcohol. This resulting strain produced 350 mg L^–1^ fatty alcohols with yields about 0.05 g/g (Akhtar et al., 2013).

The fatty acid biosynthetic pathway of *E. coli* normally yields primarily C12–C18 acyl chain molecules thus production of shorter carbon chain lengths relies on the introduction of heterologous enzymes. Production of medium chain length fatty alcohols (C6–C12) has been explored through the use of heterologous plant thioesterases, such as *Umbellularia californica* and *Cuphea hokeriana* that show preference toward C12 and C14 acyl-ACP respectively, to create C12 and C14 fatty alcohols reaching titers of up to 1.65 g L^–1^ (Steen et al., 2010; Youngquist et al., 2013). Capitalizing on the substrate preferences of the enzymes, Zheng et al. (2012) explored different combinations of fatty acyl reductases (*A. baylyi acr1*, *A. thaliana CER4*, *S. Chinensis FAR*) and fatty acyl-CoA synthetases (*E. coli fadD*, *B*. *subtilis yngL*, and *S*. *cerevisiae FAA2*) to engineer strains capable of high specificity production of C12 and C14 (*BTE* + *acr1* + *fadD*) at 449.2 mg L^–1^ or 101.5 mg L^–1^ C16 and C18 (*‘tesA* + *FAR* + *fadD*) alcohols respectively (Zheng et al., 2012). Hernandez Lozada et al. (2020) employed an acyl-ACP thioesterase derived from *Cuphea palustris CpFatB1*^∗^ engineered for increased activity and improved specificity toward C8 substrates coupled with an acyl-CoA synthetase from *Mycobacterium tuberculosis MtFadD6* and *MaFAR* to produce octanol from glycerol at a titer of 1.3 g L^–1^ and yield of 0.064 g g^–1^ (Hernandez Lozada et al., 2020).

Anaerobic production of medium chain alcohols has also been investigated through the engineering of a β-reduction pathway, essentially a reversal of the β-oxidation pathway (Mehrer et al., 2018). In principle such a pathway has an increased theoretical yield and lower ATP requirement since ATP is not required for synthesis of malonyl-CoA or for the activation of fatty acids to acyl-CoA. Expression of a heterologous *trans*-2-enoyl-CoA reductase (Ter), thiolase (FadA) and β-hydroxy-acyl-CoA reductase (FadB) allow synthesis of an acyl-CoA chain that can then undergo reduction to yield an alcohol. This strategy yielded a collection of alcohols ranging from 1-hexanol (C6) to 1-hexadecanol (C16) with 1-octanol and 1-decanol being the most prevalent. The identity of the FadAB complex influenced the dominant chain length that was produced. The highest performing strain produced alcohols at titers of 1.8 g L^–1^ with an apparent molar yield of 0.2 g g^–1^ glucose consumed (52% of theoretical yield) (Mehrer et al., 2018).

Cyanobacteria have also been explored as chassis for medium chain fatty alcohol biosynthesis. Genes encoding the key components of the 1-octanol pathway previously used in *E. coli* (Akhtar et al., 2015) including the *MmCAR*, *B. subtilis Sfp* and *A. tetradius tes3* were expressed in a *Synechocystis* sp. PCC 6803 lacking acyl-ACP synthetase. Engineering of optimal promoter and RBS sequences allowed production of 100 mg L^–1^ 1-octanol and 1-decanol. Unfortunately, these strains exhibited genetic instability when serially cultured (Yunus and Jones, 2018).

A different approach to using *E. coli* as a biocatalyst for fatty alcohol synthesis employed surface display of a lipase, CAR and Ahr (Xu et al., 2019). Engineering of a cohesin-dockerin scaffold allowed for optimization of the spatial arrangement of the displayed enzymes and allowed conversion of triglyceride feed stock into fatty alcohol with a reported 73% conversion rate and maximum titer of 65 g L^–1^ (Xu et al., 2019). Summaries of fatty alcohol production by engineered *E. coli* and cyanobacteria strains can be found in Tables 2 and 3 respectively.

**TABLE 3:** Fatty alcohol production in cyanobacteria.

**Properties**	**Host**	**Product forming enzyme**	**Mutations**	**Growth conditions**	**Yield**	**Titer (μg L^–1^)**	**Productivity (mg L^–1^h^–1^)**	**References**
Even (C_2n_) chain C16, C18	*Synechocystis sp*. PCC6803	S. *chinensis FAR*	-	BG-11, 30-50 μE m^–2^ s^–1^	9.73 μg OD^–1^ L^–1^	200.4	ND	Tan et al., 2011
	*Synechocystis sp*. PCC6803	2 x *S. chinensis FAR*, *1x At FAR2*	Δ*agp*, Δ*phaAB*	BG-11, 30-50 μE m^–2^ s^–1^	761 μg g DCW^–1^	ND	ND	Qi et al., 2013
	*Synechocystis sp*. PCC6803	*MaFAR*	Δ*ado*, Δ*aar*	BG-11, 30-50 μE m^–2^ s^–1^	2870 μg g DCW^–1^	ND	ND	Yao et al., 2014
	*Synechocystis sp*. PCC6803	*MaFAR*	Δ*plsX*	BG-11 25 mM HEPES, 60 μE m^–2^ s^–1^	∼11000 μg g DCW^–1^	∼2000	9000 μg g DCW^–1^ day^–1^	Kaczmarzyk et al., 2018
*C8, C10	*Synechocystis*	*MmCAR*, *BsSFP*, *TES3*	Δaas	BG-11 60 μE m^–2^s^–1^		100 mg L^–1^	0.416 μg L^–1^ h^–1^	Yunus and Jones, 2018

*Strain was specifically engineered for production of medium chain fatty alcohols, 1-octanol and 1-decanol. ND, Not Determined.

#### Production of Odd Chain Length and Branched Chain Fatty Alcohols

Fatty alcohols produced in *E. coli* via the *de novo* fatty acid biosynthesis pathway are exclusively of even carbon (C_2n_) chain length with the majority being C14:0, C16:0, C16:1, C18:1. However, fatty alcohols desirable for commercial applications are more diverse in structure and composed of both odd and even number of carbons as well as linear and branched chains. Controlling these parameters increases the range of applications for microbial fatty alcohols.

Production of odd chain-length fatty alcohols from fatty acid precursors has been demonstrated in *E. coli* using bacterial codon-optimized rice α-dioxygenase (*αDOX*) and *E. coli* Ahr (Cao et al., 2015). *αDOX* oxidizes long and medium-chain C_n_ fatty acids to 2-hydroperoxy fatty acids (C_n_) that are spontaneously decarboxylated to C_n–1_ fatty aldehydes. Interestingly, unlike the other FALDRs, *αDOX* does not require a reductant supply. After fine tuning of *‘tesA*, *αDOX*, AHR (*yjgB*) and *fadR* expression, the best strain produced a final titer of 1.95 g L^–1^ odd chain fatty alcohols (C15, C17) at a yield of 0.019 g g^–1^ glycerol. Similarly synthesis of branched chain fatty alcohols has been achieved by the expression of *B. subtilis alsS*, *E. coli ilvCD*, *B. subtilis bkd* and *lpdA* (lipoyl ligase) to generate branched chain α-keto acyl-CoA that can be used as primers for fatty acid biosynthesis. The resulting strain with *MaFAR*, *fadR* (overexpression) and Δ*ldhA* generated ∼170 mg L^–1^ branched chain fatty alcohol (∼80% of total fatty alcohol in shake flask) and 350 mg L^–1^ (50% of total fatty alcohol produced) in fed batch cultivation. This work opens the road for tailored conversion of carbon sources into drop-in chemical commodities.

### Engineering Fatty Alcohol Production in *Saccharomyces cerevisiae*

Like *E. coli*, the model yeast, *S. cerevisiae*, has been used extensively for research into the production of fatty acid-based chemicals and in particular, fatty alcohols. As a production chassis, *S. cerevisiae* offers the advantage of being a GRAS (generally regarded as safe) organism with an extensive genetic toolkit and well-studied biochemical pathways. It is very robust and tolerant of the harsh conditions that can be experienced during industrial scale cultivations. Further, existing bioethanol production facilities can be retrofitted to allow for production of different chemicals using *S. cerevisiae* and phage contamination, which can plague prokaryotic fermentations, is avoided by using a eukaryotic host. Here, we focus on the production of C16 and C18 chain length fatty alcohols in *S. cerevisiae*, considering (i) the enzymes that have been utilized for fatty alcohol production, (ii) metabolic engineering strategies that have been employed and (iii) attempts to take advantage of compartmentalization in cellular organelles to improve production and diversity of fatty alcohol products.

#### Enzyme Selection

*S. cerevisiae* does not encode endogenous enzymes catalyzing fatty alcohol production and thus heterologous enzyme expression is required. FARs from *Mus musculus* (*Mm*Far1), *Tyto alba* (*Ta*Far1), and *M. aquaeolei* (*MaFAR*) as well as *M. marinum* CAR, feature prominently in the *S. cerevisiae* literature (Tables 1, 4). The pioneering work engineering *S. cerevisiae* for fatty alcohol production expressed *MmFAR1* to directly catalyze the conversion of fatty acyl-CoA released from the FAS complex to fatty alcohols resulting in an initial titer of 47.4 mg L^–1^ predominantly C16:0 alcohols (Runguphan and Keasling, 2014). Subsequent expression of *TaFAR*1 resulted in comparable levels of fatty alcohols, producing 22 mg L^–1^ and 45 mg L^–1^ respectively (Feng et al., 2015; Tang and Chen, 2015). Expression of the bifunctional *MaFAR* has proven to be much less effective (<5 mg L^–1^) in *S. cerevisiae* than *TaFAR1* and *MmFAR1* (Runguphan and Keasling, 2014; Feng et al., 2015; Zhou et al., 2016b). This is curious as *MaFAR* effectively produces fatty alcohols in oleaginous yeasts (see Engineering Fatty Alcohol Production in Oleaginous Yeast). Although several groups have reported fatty alcohol production with these FARs, direct comparison of the true effectiveness of the enzymes is difficult owing to differences in promoters, auxotrophic background strains, feedstock utilized and culture conditions. A direct comparison of *MmFAR1* and *TaFAR1* under control of the *TEF1* promoter found that *TaFAR1* produced 60% of the fatty alcohols of *MmFAR1* and expression of *MaFAR* resulted in barely detectable levels of production (d’Espaux et al., 2017). Dosage of the FAR employed may be as important as the source of the enzyme. A 16-fold increase in fatty alcohol accumulation was achieved by integrating a second copy of *MmFAR* with a strong promoter into a fatty alcohol producing strain (d’Espaux et al., 2017). Integration of four copies of the *Marinobacter algicola* FAR (*MalFAR*) into the genome of an *S. cerevisiae Δpex10 Δhfd1* strain was found to produce ∼ 105 mg L^–1^ fatty alcohols, however, titers associated with a single copy of this FAR in an unmodified strain were not reported (Dahlin et al., 2019).

**TABLE 4:** Fatty alcohol production (C16 and C18) in *S. cerevisiae.*

**Background strain**	**Enzyme**	**Genotype (strain name)**	**Titer (shake flask) mg L^–1^**	**Titer (fed batch) mg L^–1^**	**Productivity (mg L^–1^h^–1^)**	**Yield (g/g) carbon**	**References**
BY4742	*MmFAR1*	pESC-P*GAL1*-mFAR1	47.4	N/A	ND	0.002	Runguphan and Keasling, 2014
BY4742	*MmFAR1*	*acc1*::P*TEF1*-*ACC1; fas1::*P*TEF1*-*FAS1; fas2::* P*TEF1*-*FAS2*, pESC-P*GAL1*-mFAR1 (WRY1)	93.4	N/A	ND	0.005	Runguphan and Keasling, 2014
BY4742	*MmFAR1*	*acc1*::P*TEF1*-*ACC1; fas1::*P*TEF1*-*FAS1*; *fas2*:: P*TEF1*-FAS2, pESC-P*GAL1*-*m FAR1/PGAL10-MaME*	98	N/A	ND	0.005	Runguphan and Keasling, 2014
BY4741	*TaFAR1*	*dga1::HIS3*; p*YES2* -P*GAL1-TaFAR1*	73	N/A	ND	0.004	Tang and Chen, 2015
BY4742	*TaFAR1*	*rpd3Δ;* pRS425-P*TEF1-TaFAR-*T*TEF1*	122.4	N/A	ND	ND	Feng et al., 2015
BY4742	*TaFAR1*	*rpd3Δ;* pRS425-P*TEF1-TaFAR-*T*TEF1-*P*PGK1-ACC1-*T*HXT7* pRS423-P*TPI1-YlACL1*-T*TPI1-*P*TEF1*-*YlACL2-*T*TEF1* (XF3)	330.2	1111	42	0.03*	Feng et al., 2015
WRY1	*MmFAR1*	1622b::P*GAL1-MmFAR1*-T*TDH1* 208a::P*TEF1-MmFAR1*-T*CYC1* YPRCd15c::PGAL1-ACC1**-T*ENO2*; *hfd1Δ adh6Δ gdh1Δ dga1Δ* 1014a::P*TEF2-OLE1*-T*ADH1 gal80Δ*::P*TDH3-GAL4* (yL434)	1200	6000	ND	0.06	d’Espaux et al., 2017
CEN.PK113-11C	*MaFAR*	*pox1Δ are1Δ are2Δ lro1Δ dga1Δ;* pYX212-(PTPI1-*MaFARper2*-pYX212t)	193	N/A	ND	0.01	Zhou et al., 2016a
CEN.PK113-11C	*MmCAR* & *MaFAR*	(FOH33) *hfd1Δ pox1Δ faa1Δ faa4Δ*, *adh6Δ::kanMX*, *gal80Δ*, *gal1/10/7Δ*::(P*GAL7-MmCA*R-T*ADH1*)+(P*GAL3-npg*A-T*FBA1*); pYX212-(P*TPI1-npgA*-T*FBA1*)+(P*TDH3-MmCAR-TADH1*)+(P*HXT7-ADH*5-T*CYC1*)+(P*TEF1-MaFAR*-pYX212t))	120	1500	ND	0.006	Zhou et al., 2016b

*Actual yields and productivity are not clear owing to the high density and initial fatty alcohol content of the starting culture. **A hyperactive *ACC1* mutant. ND, Not Determined. N/A, Not Applicable.

Alternatively, heterologous *MmCAR* expression, combined with overexpression of endogenous alcohol dehydrogenases, has also been used for fatty alcohol production in *S. cerevisiae* (Zhou et al., 2016a,b; Teixeira et al., 2017). Expression of *MmCAR* combined with alcohol dehydrogenase *ADH5* yielded fatty alcohol production 61.2 mg L^–1^ (Zhou et al., 2016b). This was further improved by expressing a combination of *MmCAR ADH5* and *MaFAR* to maximize conversion of fatty acids and acyl-CoA to fatty alcohols (Zhou et al., 2016b; Teixeira et al., 2017). An alternate two-enzyme route utilizing an aldehyde forming fatty acyl-CoA reductase from *A. baylyi* (*Ab*Acr1) or the aldehyde forming fatty acyl-ACP/-CoA reductase from *S. elongatus* (*Se*Aar) has also been described (Zhou et al., 2016b).

#### Modulating Metabolism

**FIGURE 4:**
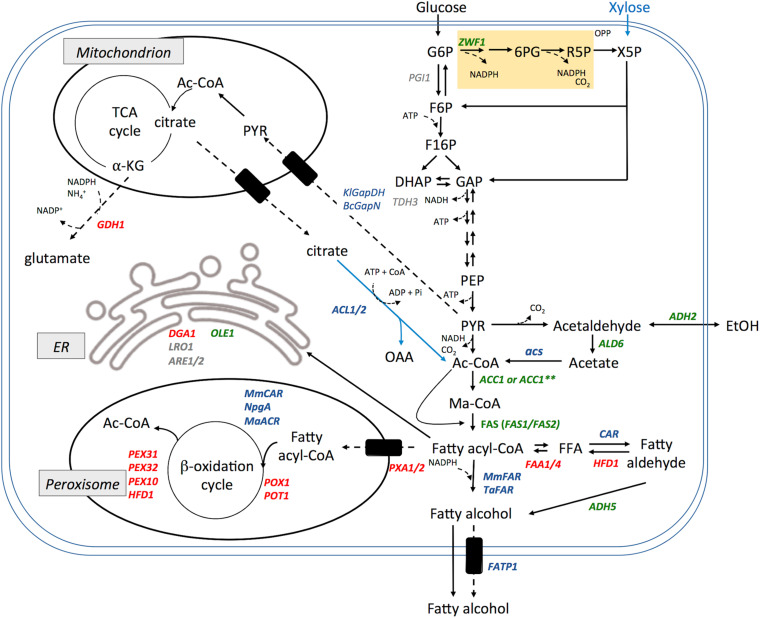
Summary of metabolic engineering performed to increase fatty alcohol production in *S. cerevisiae*. Heterologous genes expressed (shown in blue), native genes overexpressed (shown in green), native genes deleted or disrupted (shown in red). Other endogenous genes that been manipulated without improving fatty alcohol production are shown in gray. Cofactors are noted in particularly salient positions but are omitted from others for clarity. Note: these enzyme combinations have not necessarily been combined into a single production strain. Abbreviation and gene names used are: G6P, glucose-6-phosphate; 6PG, 6-phosphogluconate; R5P, ribulose-5-phosphate; X5P, Xylulose-5-phosphate; OPP, oxidative pentose phosphate pathway; F6P, fructose-6-phosphate; F16P, fructose-1,6-bisphosphate; DHAP, dihydroxyacetone phosphate; GAP, glyceraldehyde-3-phosphate; PEP, phosphoenolpyruvate; PYR, pyruvate; Ac-CoA, acetyl Co-enzyme A; Ma-CoA, Malonyl CoA; OAA, oxaloacetate; αKG, α-ketoglutarate; FFA, free fatty acid; FAS, fatty acid synthase; ER, endoplasmic reticulum; *ZWF1*, Glucose-6-phosphate dehydrogenase; *PGI*, phosphoglucose isomerase; *TDH3*, Glyceraldehyde-3-phosphate dehydrogenase; *KlGapDH*, glyceraldehyde-3-phosphate dehydrogenase from *Kluyveromyces lactis*; *BcGapN*, non-phosphorylating glyceraldehyde-3-phosphate dehydrogenase from *Bacillus cereus*; *ADH2*, alcohol dehydrogenase 2; *ALD6*, aldehyde dehydrogenase; acs, acetyl CoA synthetase; *ACC1*, acetyl-CoA carboxylase; *ACC1*^∗∗^, A hyperactive *ACC1* mutant; *ACL1/2*, ATP-citrate lyase; *GDH1*, glutamate dehydrogenase; *DGA1*, diacylglycerol acyltransferase; *LRO1*, acyltransferase; *ARE1/2*, Acyl-CoA:sterol acyltransferase; *OLE1*, Δ9 fatty acid desaturase; *HFD1*, fatty aldehyde dehydrogenase; *FAA1/4*, Long chain fatty acyl-CoA synthetase; *ADH5*, alcohol dehydrogenase isozyme 5; *PXA1/2*, peroxisomal ABC-transporter; *POX1*, fatty acyl-CoA oxidase; *POT1*, Peroxisomal Oxoacyl Thiolase; *PEX10/31/32*, peroxisomal membrane proteins; FATP1, human free fatty acid transporter; *MmCAR*, *Mycobacterium marinum* carboxylic acid reductase; NpgA, activator protein from *Aspergillus nidulans*; *MaFAR*, *Marinobacter aquaeolei* VT8 acyl-CoA reductase; *TaFAR1*, *Tyto alba* fatty acyl-CoA reductase 1; *MmFAR1*, *Mus musculus* fatty acyl-CoA reductase 1.

Although *S. cerevisiae* is a well-characterized and highly facile model organism, its fermentative metabolism directs carbon from sugars toward energy production by glycolysis leading to the generation of ethanol (Crabtree positive) with only modest amounts channeled toward acetyl-CoA. The lipid biosynthetic pathway in this organism predominantly produces C16 and C18 chain-length fatty acyl-CoA that are directed toward membrane synthesis. Not surprisingly, *S. cerevisiae* naturally accumulates only a small amount of triacylglycerol and thus its metabolic pathways require substantial rewiring to achieve high levels of fatty acyl-CoA or fatty acids to direct toward fatty alcohol biosynthesis. An overview of metabolic engineering performed to increase fatty alcohol production by *S. cerevisiae* is shown (Figure 4).

Increasing flux through the fatty acid synthesis pathway can be limited, at least in part, by the availability of the initial substrate, acetyl-CoA. Metabolic engineering to increase cytosolic acetyl-CoA availability has been extensively pursued and reviewed (Krivoruchko et al., 2015). One approach to increasing acetyl-CoA supply for fatty alcohol biosynthesis in *S. cerevisiae* is to mimic the process naturally employed by oleaginous organisms and express an ATP-dependent citrate lyase (ACL). Feng et al. (2015) co-expressed *ACL1* and *ACL2*, from either *A. thaliana* or *Y. lipolytica* in a fatty alcohol producing *S. cerevisiae* strain resulting in a 55 and 136% increase in 1-hexadecanol production, respectively achieving up to 330 mg L^–1^ titer in shake flask culture (Feng et al., 2015). A further advance in increasing acetyl-CoA supply was achieved by generating an optimized citrate lyase cycle composed of ACL, malic enzyme and malate dehydrogenase (Zhou et al., 2016b).

As flux through the fatty acid synthesis pathway is relatively low, increasing the availability of acyl-CoA substrates has been a major focus of metabolic engineering for fatty alcohol production in *S. cerevisiae* (Figure 4). Overexpression of *ACC1* has been found to result in an up to a 56% increase in fatty alcohols (Runguphan and Keasling, 2014; Feng et al., 2015). Further, introducing a second copy of Acc1 that was modified by mutating phosphorylation sites (S^659^A and S^1157^A) to eliminate post-translational regulation has been found to increase fatty alcohol production by 2.6-fold (d’Espaux et al., 2017). However, it is unclear whether the observed increase in fatty alcohols was due to the increased copy number or decreased regulation of Acc1. Deletion of the *SNF1* gene encoding the kinase that down regulates Acc1 activity lead to a decrease in fatty alcohol production suggesting that regulation by Snf1 is not a limiting factor in fatty alcohol production in *S. cerevisiae* (Feng et al., 2015). Overexpression of *FAS1* and *FAS2* in a fatty alcohol producing strain has also been reported to increase flux through the FAS pathway leading to a 65% increase in fatty alcohol biosynthesis (Runguphan and Keasling, 2014). Overexpression of the Δ9-desaturase gene *OLE1* has also been used successfully to drive flux through the FAS pathway and increase fatty alcohol production by decreasing feedback inhibition exerted by saturated fatty acyl-CoA (Lust and Lynen, 1968; d’Espaux et al., 2017).

Fatty acids are inherently more stable than fatty acyl-CoA and can accumulate to higher intracellular concentration making them a potentially better precursor for fatty alcohol synthesis. Increased accumulation of free fatty acids has been achieved by deletion of acyl-CoA synthetases *FAA1* and *FAA4* (Scharnewski et al., 2008). In this strain background expression of acyl-aldehyde generating *MmCAR* and its activating subunit 4’ phosphopantetheinyl transferase resulted in 24.3 mg L^–1^ fatty alcohol production (Tang et al., 2017). Additional expression of a thioesterase and deletion of acyl-transferase *DGA1* lead to 31.2 mg L^–1^ fatty alcohols (Tang et al., 2017). Further strain engineering to delete *POX1* and aldehyde dehydrogenase *HFD1* coupled with over expression of *ADH5* lead to further increase in fatty alcohol titer. However, the use of this strategy resulted in accumulation of excess acyl-aldehyde or fatty acids that were secreted from the cells thus lowering the yield of fatty alcohol products suggesting that final conversion by the CAR or ADH enzymes were rate limiting (Teixeira et al., 2017). Expression of a combination of *MmCAR* and *MaFAR* enzymes reduced accumulation of acyl-aldehyde intermediates and increased fatty alcohol production to 52 mg L^–1^ (Teixeira et al., 2017). The highest reported fatty alcohol titers generated through the fatty acids route were achieved with a *Δfaa1ΔΔfaa4 Δhfd1 Δpox1 Δadh6 Δgal80* strain over producing genome integrated copies of *MmCAR* and its activator *Aspergillus nidulans npgA*, along with plasmid borne copies of *MmCAR*, *npgA* and *MaFAR* 120 mg L^–1^ in shake flask and 1.5 g L^–1^ in fed batch culture (Zhou et al., 2016b). Despite the high titer achieved with this strain the yield of 0.005 g g^–1^ glucose, was significantly below the maximum theoretical yield of 0.35 g g^–1^ glucose (Zhou et al., 2016b).

The effect of transcriptional regulators on fatty alcohol production in *S. cerevisiae* has been investigated by deletions of genes coding for repressors of phospholipid biosynthesis. It was suggested that deletion of *RPD3*, *MOT1* and *OPI1* had positive effects on fatty alcohol biosynthesis (Feng et al., 2015). However, more extensive investigation found no substantial gains in fatty alcohol production were achieved by installing these deletions (Sheng et al., 2016; d’Espaux et al., 2017).

Fatty acyl-CoA has multiple cellular fates including major forms of lipids, such as triacylglycerols (TAGs), sterol esters (SEs), and membrane phospholipids. Routing acyl-CoA toward synthesis of these lipids competes with FAR or CAR for the availability of substrates to catalyze conversion to fatty alcohols (Tehlivets et al., 2007). Therefore, deletions of genes involved in these pathways have been investigated for their effects on fatty alcohol production. Eliminating triglyceride production through the deletion of *DGA1*, the terminal enzyme and rate-limiting step in TAG synthesis, has been most successful. Deletion of *DGA1* in a *TaFAR1* expressing strain increased fatty alcohol titer 1.7-fold from 26 to 45 mg L ^–1^ (Tang and Chen, 2015) and in a strain already engineered for increased fatty alcohol production, a 6.8-fold increase in fatty alcohols was observed (d’Espaux et al., 2017). However, deletion of other genes involved in the synthesis of TAGs and SEs, *LRO1Δ*, *ARE1Δ*, and *ARE2Δ* had no effect on fatty alcohol production (d’Espaux et al., 2017).

Even though eliminating the degradation of fatty acyl-CoA through the β-oxidation pathway has been extensively explored, only marginal effects on the fatty alcohol production have been observed in *S. cerevisiae*. Deletion of the peroxisomal fatty acyl-CoA importer genes *PXA1* and *PXA2* have no effect on fatty alcohol production (Runguphan and Keasling, 2014; d’Espaux et al., 2017). Deletion of *POX1*, an acyl-CoA oxidase, has reported mixed results. Deletion of *POX1* in an *ACC1* and *MmFAR1* overexpressing strain resulted in a modest (∼20%) increase in fatty alcohol production but provided no improvement when installed in a high fatty alcohol producing strain (Runguphan and Keasling, 2014; d’Espaux et al., 2017). Feng et al. deleted both *POX1* and the peroxisomal oxoacyl thiolase, *POT1* and observed a drastic decrease in fatty alcohol production (Feng et al., 2015). Use of *pox1*Δ strains is frequently reported for producing fatty alcohols from free fatty acids using both *MmCAR* and *MaFAR* and does not appear to be detrimental (Zhou et al., 2016a,b; Teixeira et al., 2017). Owing to significant glucose repression of other catabolic pathways it is likely that so long as sufficient glucose is available *POX1* expression is repressed and β-oxidation of acyl-CoA will present a minor limitation for fatty alcohol biosynthesis and accumulation (Wang et al., 1994).

Additionally, studies of carbon flux in *S. cerevisiae* have implicated glutamate, glycerol and ethanol as major sinks for the catabolized carbon (d’Espaux et al., 2017). Deletion of the glutamate dehydrogenase gene *GDH1* increased fatty alcohol titers by 2.7-fold while deleting the endogenous aldehyde dehydrogenase *HFD1* and alcohol dehydrogenase *ADH6* increased fatty alcohol titers by 2.6-fold and 1.5-fold in a fatty alcohol producing strain (d’Espaux et al., 2017). Previous work also demonstrated that deletion of *HFD1* was critical to maximize production of fatty alcohols in strains expressing *S. elongatus* aldehyde forming reductase, as this modification prevented the reversal of fatty aldehydes back into fatty acids (Buijs et al., 2015). These results indicate that competitive pathways, including the production of free fatty acids, can act as a significant diversion to incoming carbon and that their ablation can increase carbon available to fatty alcohol synthesis.

Proteomic studies and carbon flux analysis revealed that the vast majority of sugar derived carbon proceeds through glycolysis with only a small flux of carbon routed through the pentose phosphate pathway (d’Espaux et al., 2017). Therefore, *S. cerevisiae* metabolism is poised for generating NADH as opposed to the NADPH required for fatty alcohols. To combat this, strategies to increase NADPH production have been tested. One strategy to increase NADPH has been the overexpression of NADP-dependent malic enzyme, which converts malate and NADP^+^ to pyruvate and NADPH, releasing carbon dioxide in the process. Using this approach, heterologous expression the malic enzyme from the oleaginous fungus *Mortierella alpina* in a fatty alcohol producing *S. cerevisiae* strain resulted in a modest increase from 93.4 mg L^–1^ to 98.0 mg L^–1^ fatty alcohol (Runguphan and Keasling, 2014). More recently d’Espaux et al. attempted to address redox optimization in a variety of ways. Their proteomic studies and carbon flux analysis revealed that large amounts of NADH are being produced through the highly expressed glyceraldehyde-3-phosphate dehydrogenase (GapDH) Tdh3. Replacement the native NADH-producing Tdh3 with either an NADPH-producing GapDH from *Kluyveromyces lactis* (KlGapDH), or a non-phosphorylating GapDH from *Bacillus cereus* (BcGapN), had no effect on fatty alcohol production. Alternatively, *PGI1* was deleted and *ZWF1* over expressed to force carbon flux through the pentose phosphate pathway to generate NADPH. However, the resulting strain grew slowly and produced very low levels of fatty alcohols (d’Espaux et al., 2017).

The highest titers of fatty alcohol achieved using *S. cerevisiae* as a production chassis have stacked 11 successful genetic modifications to produce a strain (yL434) that saw a 43-fold increase in fatty alcohol production over a starting strain whose only modification is expression of *MmFAR1*. The strain incorporates overexpression of *ACC1*, *FAS1*, *FAS2*, *OLE1*, a hyperactive *ACC1*, two genome-integrated copies of *MmFAR1*, deletions of *HFD1*, *ADH6*, *GDH1*, *DGA1* and *GAL80*. The resulting strain generated titers of 1.2 g L^–1^ in shake flask and was scaled up using alternate feeding strategies to produce more than 6.0 g L^–1^ in a bioreactor (d’Espaux et al., 2017).

*S. cerevisiae* produces predominantly C16 and C18 chain length fatty acids through the endogenous FAS biosynthetic pathway thus, most genetic engineering efforts reported have been focused on the production of mixtures of 1-hexadecanol and 1-octadecanol. However, *S. cerevisiae* has also been engineered for production of 1-octanol (C8:0) through expression of a modified FAS pathway and MmCAR resulting in 49.5 mg L^–1^ 1-octanol (Henritzi et al., 2018). A titer of 1.3 g L^–1^ medium chain fatty alcohols (MCFOH), 1-decanol (C10:0), 1-dodecanol (C12:0), and 1-tetradecaonol (C14:0) were produced by *S. cerevisiae* through targeting *TaFAR* to the peroxisome coupled with elevated *ACC1* expression (Sheng et al., 2016). Recently, an engineered version of *MmCAR* and its activator *NpgA* were expressed in a background strain over expressing *AnACL, MmACL, CTP1, FAS*^∗^ (engineered endogenous version for production of medium chain fatty acyl-CoA), ‘*MDH3* and *RtME*, to increase acetyl-CoA production and harboring, *Δpox1*, *Δhfd1* to inhibit product reversal resulting in 2.8 fold higher MCFOH than non-engineered enzyme in the same background strain. Deletion of the MCFA exporter *TPO1* was found to further increase yield to 252 mg/L (Hu et al., 2020). Synthesis of 83.5 mg L^–1^ very long chain fatty alcohols including 1-eicosanol (C20:0) and 1-docosanol (C22:0) was achieved through expression of a *Mycobacterium vaccae* FAS enzyme, over expression of endogenous fatty acid elongases *ELO1* and *ELO2* along with *AtFAR1* (Yu et al., 2017). A similar strategy employed a heterologous elongase from *Crambe abyssinica* and expression of *MaFAR* to produce 1 mg g^–1^ DCW C20 and C22 fatty alcohols (Wenning et al., 2019). Biosynthesis of 20.9 mg L^–1^ odd chain length fatty alcohols (C13:0, C15:0) has also been demonstrated in *S. cerevisiae* engineered to over produce fatty acids coupled with expression of *Oryza sativa αDOX* and over expression of endogenous alcohol dehydrogenases (Jin et al., 2016).

#### Cellular Compartmentalization of Fatty Alcohol Biosynthesis

Eukaryotic cells harbor membrane bound organelles that allow for compartmentalization of biosynthetic or degradative processes. Sequestering biosynthetic pathways within an organelle has the potential to increase substrate channeling and limit the loss of intermediates from the pathway. Use of pathway compartmentalization targeting fatty alcohol biosynthesis to the peroxisome has been employed as an alternate strategy for the production of fatty alcohols (Sheng et al., 2016; Zhou et al., 2016a). For the production of medium chain length fatty alcohols *TaFAR1* was expressed with a peroxisomal signal sequence in conjunction with overexpression of *ACC1* and *PEX7*, resulting in a strain producing over 1.3 g L^–1^ of medium and long chain fatty alcohols in fed-batch culture (Sheng et al., 2016). Similarly, in a strain engineered for increased FFA production, targeting *MaFAR* to the peroxisome rather than the cytosol yielded a 63% improvement in fatty alcohol titer (Zhou et al., 2016a). There is some evidence that this could be further improved by expression of *MmCAR* in the peroxisome and mutations to increase peroxisome biogenesis (Zhou et al., 2016a). A further potential benefit to sequestering a fatty alcohol biosynthesis pathway to the peroxisome is that this pathway increased the yield of unsaturated and short chain fatty alcohols, likely due to capture of intermediates from β-oxidation. Additionally, fatty alcohol synthesis continued after glucose had been exhausted from the growth medium, likely reflecting capture of acyl-CoA generated by lipolysis and transported to the peroxisome for breakdown by β-oxidation (Zhou et al., 2016a).

### Engineering Fatty Alcohol Production in Oleaginous Yeasts

Oleaginous yeasts have metabolic pathways that allow a high flux of incoming carbon to be directed toward acyl-CoA production (Fillet et al., 2015) and are defined by their ability to accumulate greater than 20% of their cell weight as lipids. This inherent capability makes them appealing as chassis for the production of oleochemicals. To date, three oleaginous yeasts, *Rhodosporidium toruloides*, *Yarrowia lipolytica*, and *Lipomyces starkeyi*, have been successfully engineered to produce fatty alcohols (Fillet et al., 2015; Rutter and Rao, 2016; Wang G. et al., 2016; Wang W. et al., 2016; Xu et al., 2016; McNeil and Stuart, 2018b). The engineering performed and outcomes achieved with oleaginous strains are summarized in Table 5.

**TABLE 5:** Fatty alcohol production in oleaginous yeasts.

**Species name**	**Background strain**	**Enzyme**	**Genotype/Mutations**	**Titer (shake flask) mg L^–1^**	**Titer (fed Batch) mg L^–1^**	**Yield (g/g) carbon**	**References**
*Rhodosporidium toluroides NS134*	CECT13085	*MaFAR* (Codon optimized)	2x P*GPD1*-maqRt-Tnos-pPGK47-G418Rt-T35S	ND	8000	0.04*	Fillet et al., 2015
*Yarrowia lipolytica*	Po1f or H222	*TaFAR1*	5x *TaFAR1 dga1Δ fao1Δ*	ND	690.21	0.004	Wang G. et al., 2016
	Po1g	*MaFAR* (Codon optimized)	none	167	N/A	0.006	Wang W. et al., 2016
	Po1g	*MaFAR* (Codon optimized)	pYLXP-*MaFAR* -*EcfadD*	205.4	2150	.0215**	Xu et al., 2016
	ATCC 201249	*MaFAR*	MaACR *Ku80Δ sct1Δ OLE1*	–	5750	.063*	Zhang et al., 2019
	L36DGA1	*MaFAR*	2x MaACR *mga2 G^643^R DGA1*	–	5800	.036	Cordova et al., 2020
*Lipomyces starkeyi*	NRRL Y-11557	*MaFAR* (Codon optimized)	P*LsPYK1-MaFAR-*T*LsGAL1*	770	N/A	0.025	Wang W. et al., 2016
	NRRL Y-11557	*MmFAR* (Codon optimized)	P*URA3*-Nat1-*TDH3*t-P*TDH3*-*mFAR1Yl-*T*URA3*	Variable dependant on culture conditions	1700	0.028	McNeil and Stuart, 2018b
	NRRL Y-11557	*6 copies MaFAR*	P*LsPYK1-MaFAR-*T*LsGAL1*	770	4200	0.028	Wang et al., 2020

* Complex medium, carbon sources not fully defined. **Seeded with 120 mL of 48 h shake flask culture. ND, Not Determined. N/A, Not Applicable.

#### Fatty Alcohol Production by *Y. lipolytica*

*Y. lipolytica* has emerged as a model oleaginous yeast and therefore has benefitted from the development of numerous genetic tools. In 2016, four separate reports of fatty alcohol production in *Y. lipolytica* were published (Rutter and Rao, 2016; Wang G. et al., 2016; Wang W. et al., 2016; Xu et al., 2016) and since 2016 four additional studies have investigated engineering *Y. lipolytica* for fatty alcohol biosynthesis (Dahlin et al., 2019; Zhang et al., 2019; Cordova et al., 2020; Holkenbrink et al., 2020).

Building on work performed in *S. cerevisiae*, integration of *TaFAR1* or *MaFAR* in *Y. lipolytica* yields C16 and C18 fatty alcohols and increased copy number of the Far enzyme leads to increased product synthesis suggesting that the Far activity may be rate limiting (Wang G. et al., 2016; Wang W. et al., 2016). These investigations determined that genetic manipulations to increase acyl-CoA availability like deletion of *DGA1* coupled with deletion of fatty alcohol oxidase *FAO1* could increase fatty alcohol accumulation. Similar to the case with *S. cerevisiae* greater than 90% of the product fatty alcohols were retained in the cells (636.9 mg L^–1^ intracellular vs 53.3 mg L^–1^ extracellular) (Wang G. et al., 2016). The addition of dodecane to the growth medium increased the proportion of fatty alcohol found extracellularly (Wang W. et al., 2016; Cordova et al., 2020). The addition of dodecane also increased the total fatty alcohol titer suggesting that retention of the product in the cells was inhibiting biosynthesis (Wang G. et al., 2016).

Further advances in engineering *Y. lipolytica* involve taking advantage of the natural lipid biosynthetic pathway of the yeast. Acyl-CoA is synthesized in the cytoplasm and a high proportion is shuttled to the endoplasmic reticulum for membrane lipid synthesis (Rasmussen et al., 1994). Xu et al. (2016) expressed *AbACR1* and *E. coli* aldehyde reductase (Ec*AHR*), targeting these enzymes to the endoplasmic reticulum to take advantage of the native flux of acyl-CoA to the ER. Fatty alcohol production (49.2 mg L^–1^) was relatively modest. Greater accumulation of fatty alcohol was observed by coupling *MaFAR* and *E. coli FadD* (203.4 mg L^–1^ (Xu et al., 2016). When cultured at 3 L scale this strain produced 2.15 g L^–1^ fatty alcohols and a yield of 0.022 g g^–1^ glucose (Xu et al., 2016). Optimizing expression of *MaFAR* by testing a variety of promoters coupled with deletion of acyl-transferases *DGA1* and *SCT1*, which compete for acyl-CoA substrate, and over expression of desaturase *OLE1* to reduce feedback inhibition of acyl-CoA synthesis achieved a production titer of 5.75 g L^–1^ (0.063 g g^–1^ glucose) fatty alcohol (Zhang et al., 2019). It is notable that the culture was performed in complex medium making it difficult to determine true yields owing to the presence of carbon sources other than the added glucose. In contrast, an investigation by Cordova et al. (2020) made use of defined medium and a strain deficient in acyl-CoA degradation (*Δpex10Δmfe1*) over expressing acyl-transferase *DGA1* and two copies of *MaFAR* to produce a final titer of 5.8 g L^–1^ of fatty alcohols (0.036 g g^–1^ glucose). The high titer was achieved in a fed batch bioreactor culture with pH and dissolved oxygen control. It is likely that greater yield could have been achieved had it been possible to employ a dodecane overlay to the culture. Additionally, it was noted that citrate accumulated in the medium suggesting that ATP-citrate lyase activity was not sufficient to capture all the citrate generated and direct it to acetyl-CoA thus limiting the substrate available for acyl-CoA synthesis (Cordova et al., 2020).

Saturated hexadecanol and octadecanol are the most common fatty alcohols produced by microbial cell factories as their sixteen and eighteen carbon acyl-CoA precursors are the most abundant in cells. Fatty alcohols with specific sites of mono-unsaturation have been produced in *Y. lipolytica*. A strain deficient in aldehyde dehydrogenases *Δhfd1*, *Δhfd4*, fatty alcohol oxidase *Δfao1* and peroxisomal biogenesis factor *Δpex10* has been engineered to produce *cis*-11-hexadecanol, an insect pheromone, at a titer of 2.75 g L^–1^ through expression of acyl-CoA desaturase from *Amyelois tansitella* and FAR enzymes from *Heliothis subflexia* and *Helicoverpa armigra*. These authors were also able to produce *cis*-9-tetradecanol by introducing a mutation into fatty acyl-CoA synthetase, Fas2^I1220F^ and expression of *D. melanogaster* acyl-CoA desaturase and *H. armigra* FAR (Holkenbrink et al., 2020). *Y. lipolytica* has been engineered to produce the medium chain fatty alcohol 1-decanol by expression of acyl-ACP-thioesterases from *A. tetadius*, *C. hookeriana*, *C. palustris*, *C. perfringens*, and *U. californica* with specificity to produce 8–10 carbon chain fatty acids and *AtFAR6* (Rutter and Rao, 2016). Combined with deletion of *PEX10* the strain could accumulate greater than 500 mg L^–1^ 1-decanol with lower levels of 1-hexadecanol and 1-octadecanol also present (Rutter and Rao, 2016).

#### Fatty Alcohol Production in *R. toruloides*

The first oleaginous yeast engineered for fatty alcohol production was *R. toruloides* (Fillet et al., 2015). A strain that had been selected and evolved to produce over 60 g L^–1^ triglycerides was engineered to express *MaFAR*. This strain (NS-134) produced the highest reported titer to date of C16–C18 fatty alcohols, with over 8 g L^–1^ and a yield of 0.04 g g^–1^ being produced in fed-batch bioreactors (Fillet et al., 2015). The actual yield is difficult to ascertain however, since the production medium included a combination of sucrose and corn steep liquor and the composition of the latter was undefined. Interestingly, the predominant fatty alcohols produced by the NS-134 strain differ from that reported in *S. cerevisiae*, *Y. lipolytica* and *L. starkeyi* with oleyl alcohol (C18:1), stearyl alcohol (C18:0) and cetyl alcohol (C16:0), representing 57, 24 and 19% of the total fatty alcohols produced (Fillet et al., 2015). The fatty alcohols produced by this strain are also predominately secreted but not until a titer of approximately 1 g L^–1^ is reached. It is likely that the detergent added to the medium (5 g L^–1^ tergitol) contributed to efficient secretion of the fatty alcohol products (Fillet et al., 2015). Further investigations demonstrated that a variety of surfactant additives improve secretion of fatty alcohol products and reduce the toxicity associated with intracellular accumulation of the fatty alcohols in engineered *R. toruloides* (Liu et al., 2020).

#### Fatty Alcohol Production in *L. starkeyi*

*L. starkeyi* has emerged as a microbial strain with outstanding potential for industrial scale production of oleochemicals (McNeil and Stuart, 2018a). The strain has been engineered to express either *MaFAR* or *MmFAR* resulting in as much as ∼750 mg L^–1^ fatty alcohol and yields of 0.028 g g^–1^ glucose in shake flask cultures (Wang W. et al., 2016; McNeil and Stuart, 2018b). A significant advantage of *L. starkeyi* over some other oleaginous yeast is that it can assimilate a wide variety of carbon feedstock and is tolerant of common inhibitory compounds in cellulosic hydrolysates (Riley et al., 2016; Xavier et al., 2017). Indeed, both the *MmFAR1* and *MaFAR* expressing strains of *L. starkeyi* produced high levels of fatty alcohols from either hexose or pentose sugars (Wang W. et al., 2016; McNeil and Stuart, 2018b). Scale up for the *MmFAR1* containing strain under optimized conditions in a 2 L bioreactor resulted in an extracellular titer of 1.7 g L^–1^ fatty alcohol produced from glucose (McNeil and Stuart, 2018b). It is notable that greater than 85% of the fatty alcohols produced were secreted into the growth medium without the addition of surfactants or a dodecane overlay. Additionally, a recent report demonstrated production of 4.2 g L^–1^ fatty alcohol from a strain harboring six-integrated copies of *MaFAR* (Wang et al., 2020). Despite the high titer reported by Wang et al., 2020 the yield of fatty alcohol remained 0.028 g g^–1^ glucose (Wang et al., 2020). No engineering of the endogenous metabolic pathways of *L. starkeyi* was performed in these strains, suggesting that with the continued emergence of genetic tools for the organism *L. starkeyi* may make an excellent chassis for industrial scale fatty acid based chemical production.

## Prospectives

Microbial production of fatty alcohol has significantly advanced since the pioneering work in *E. coli* by Steen et al. (2010). However, production still lags significantly behind theoretically maximum yields. In principle it should be possible to achieve ∼0.34 g of C16 fatty alcohols per gram of glucose feedstock, yet in practice levels of production in *E. coli* are less than half that, while in yeasts, production lags even further behind with the highest yields being 0.02–0.04 g g^–1^ glucose. Maximum yields can only be achieved when synthesis is decoupled from biomass accumulation. Thus, ultimately yields will always be submaximal owing to the need to channel a portion of feedstock carbon to biomass accumulation. A challenge moving forward will be to optimize cultivation conditions to maximize the flux of feedstock carbon to products and minimize the flux toward biomass.

Decoupling cell growth from oleochemical product synthesis can be achieved through either process engineering or genetic engineering of the production strain. From a process engineering standpoint, cultivation in medium with limiting amounts of a key non-carbon nutrient such as phosphorous, sulfur or nitrogen can be used to limit biomass accumulation and induce a switch toward lipogenesis (Ratledge and Wynn, 2002; Wu et al., 2011; Wang et al., 2018). Elevated salinity can induce lipogenesis in some algal species (Mao et al., 2020). Two-stage cultivations where biomass is initially accumulated in the presence of all required nutrients followed by a second phase in high carbon, limited nitrogen medium has been employed to achieve high titres of lipids (Karamerou and Webb, 2019). Fed batch cultivations achieve the same end but require only a single vessel (Wang et al., 2020).

Alternatively, the problem of decoupling cellular growth from oleochemical production can be addressed through genetic engineering. Transcriptomic analysis has revealed extensive genome-wide changes in gene expression of *R. toruloides* and *Y. lipolytica* cultured in nitrogen-limited medium (Zhu et al., 2012; Dahlin et al., 2019). Therefore, mimicking the effects of nutrient limitation through genetic manipulation would be complex and challenging and may not be the most direct way of approaching this problem through genome engineering. However, metabolic engineering has been applied to decouple biomass accumulation and wax-ester synthesis in *Acinetobacter baylyi* by placing the *aceA* gene encoding isocitrate lyase under the control of a repressible promoter so that in the presence of acetate as a carbon source *aceA* expression could be inactivated to divert carbon from biomass to wax ester synthesis without limiting nitrogen (Santala et al., 2018; Luo et al., 2020). A post-translational regulation strategy has been applied to *E. coli* to decouple biomass accumulation and poly(3-hydroxybutyrate) synthesis (Durante-Rodríguez et al., 2018). Mutations that delay or arrest cell cycle progression (Madeira et al., 2019; Torres-Romero et al., 2020) have also been found to increase cellular oil production and therefore stand to be reasonable targets for genetic engineering in other organisms. These achievements suggest that at least some host organisms may be amenable to engineering strategies that effectively redirect carbon flux away from biomass accumulation and toward product synthesis to maximize fatty alcohol yields.

**Increasing fatty alcohol synthesis and yields:** Engineering carbon flux toward fatty alcohol synthesis may still benefit from identification of bottlenecks and improvement of specific enzyme activities. Despite the aforementioned limitations there is still substantial opportunity to engineer an increased flux of carbon toward fatty alcohol products. In all microbial organisms used as production chassis for fatty alcohols the production of fatty acids as products exceeds fatty alcohol synthesis (Table 6). In oleaginous yeast producing fatty alcohol, citrate accumulation is observed and a substantial amount of fatty acid and triglyceride accumulates suggesting limitations in both the acyl-CoA synthesis pathway and the activity of the FAR or CAR encoded enzymes (Wang W. et al., 2016; Xu et al., 2016; Zhou et al., 2016b; Teixeira et al., 2017; Dahlin et al., 2019; Cordova et al., 2020). This may be improved to some degree by increasing copy number and promoter strength of the FAR or CAR genes, increasing the availability of acyl-CoA substrate to the *FAR* or *CAR* enzymes, or through protein engineering to increase the catalytic activity of the enzyme. Current methods of coupling machine learning principles such as “Design-Build-Test-Learn (DBTL)” with synthetic biology are likely to lead to unexpected approaches to metabolic engineering yielding improvements in fatty alcohol biosynthesis (Opgenorth et al., 2019).

**TABLE 6:** Summary of free fatty acid production by selected microbial species.

**Organism**	**Titer (g L^–1^)**	**Yield (g g^–1^ glucose)**	**References**
*E. coli*	7	0.28	Dellomonaco et al., 2011
*E. coli*	17.5	0.13	Jawed et al., 2016
*R. opacus*	50.2	0.25	Kim et al., 2019
*S. elongatus*	0.64	0.38*	Kato et al., 2017
*S. sp. PCC 11901*	1.54	ND	Włodarczyk et al., 2020
*S. cerevisiae*	33.4	0.07	Yu et al., 2018
*Y. lipolytica*	10.4	0.087	Ledesma-Amaro et al., 2016

*Yield is refers to grams of product per gram of glucose feedstock with the exception of cyanobacteria where yield is reported as grams of product per gram dry cell weight.

**Decreasing feedstock cost:** Feedstock cost relative to product yields and value can be a limiting factor in achieving a commercially viable microbial cell production process. Any process using microbial cells to produce chemicals would be more sustainable if abundant low-value lignocellulosic or hemicellulosic sugars derived from agricultural or forestry waste were used as feedstock. The vast majority of cultivations using engineered *S. cerevisiae* for fatty alcohol production have employed glucose as a carbon source. *S. cerevisiae* has some limitations in this regard as it does not have a robust ability to metabolize pentose sugars and displays strong glucose repression of alternate carbon metabolism. A xylose utilization pathway was introduced into a fatty alcohol producing strain allowing limited conversion of xylose to 1-hexadecanol (Guo et al., 2016). *L. starkeyi*, *R. toruloides* and *E. coli* are capable of utilizing a wider range of feedstock including the pentose sugars that are the dominant monosaccharides in hemicellulose but engineering improved utilization of di and polysaccharides may prove beneficial. The feasibility of using cellulosic sugars for fatty alcohol production was demonstrated by using ionic liquid treated, enzymatically saccharified switch grass and sorghum to produce 0.7 g L^–1^ fatty alcohols (d’Espaux et al., 2017). The utilization of pentose sugars from a mixed carbon feedstock has yet to be demonstrated for fatty alcohol production and considering that most biomass sources contain substantial lignin and pentose sugars this will be a goal for future engineering efforts. Similarly, there will be economic benefits to improving tolerance of production strains to inhibitory compounds including furfural, hydroxymethylfurfural, and organic acids formed during pre-processing of cellulosic and hemicellulosic biomass for feedstock. Crude glycerol derived from biodiesel production could also be employed as a low-cost feedstock for fatty alcohol production.

Alternatively, cyanobacteria take advantage of abundant low-cost CO_2_ as feedstock but remain challenged as a cell factory by modest productivities. Other low-cost carbon feedstocks such as methane and methanol, abundant industrial by products, have potential for conversion to high-value chemicals. Methanol may be a more suitable feedstock from the perspective of bio-manufacturing/biorefining. The liquid form of methanol simplifies storage and transportation of the material and relative to the gas form methane the liquid form allows improved mass transfer in fermentation reactions allowing higher productivity (Pfeifenschneider et al., 2017; Zhou et al., 2018). Despite this potential, methanotrophic and methyltrophic organisms have yet to be exploited for fatty alcohol biosynthesis.

**Increased product diversity:** The highest product titers to date have been achieved for C16 and C18 alcohols, hexadecanol and octadecanol. These saturated fatty alcohols have wide industrial applications but currently they are readily synthesized in large volume as relatively low-cost commodity chemicals from palm oil and from petrochemical feedstock. Owing to feedstock and production cost coupled to modest product yields it will be a challenge to achieve an economically viable process for microbial synthesis of these commodity chemicals. In contrast, microbial cell factories have a profound advantage in the ability to produce higher value short chain alcohols, branched chain alcohols and alcohols with specified or multiple sites of desaturation. Additionally, coupling the biosynthesis of higher value chemicals with production of fatty alcohols and other oleochemicals in a biorefinery process may be a mechanism to improve the economics of production and maximize carbon usage.

**Improved product secretion**. The majority of fatty alcohols produced in *E. coli*, *S. cerevisiae* and *Y. lipolytica* accumulate intracellularly. This becomes self-limiting, likely owing to a combination of product inhibition, and toxicity caused by interference with membrane structure and function (Dahlin et al., 2019). Additionally, it increases the cost of downstream processing. A widely employed solution is the use of a two-phase culture with dodecane layered over the growth medium to act as a hydrophobic sink that segregates the product from the cells and growth medium (Steen et al., 2010). While this strategy has proven effective it increases the complexity of large-scale production and increases the cost of the process. Elevated product secretion through installation of specific efflux pumps or transporters appears to be an elegant solution. Fatty alcohol secretion from *S. cerevisiae* has been achieved through expression of a human free fatty acid transporter FATP1 (Hu et al., 2018). However, it is unclear whether or not efficient transporters can be expressed without adding further metabolic burden to the host chassis. Modification of the plasma membrane composition of production hosts also has potential but currently the dynamics of fatty alcohol transit through the plasma membrane are not well understood. Considering the potential benefit of achieving efficient product secretion it is likely that this will continue to be an avenue of active investigation.

**Expanding the range of host strains and genetic tools for their improvement**. Numerous microbial species with outstanding potential for the synthesis of fatty alcohols and oleochemicals have emerged. One such example is the oleaginous bacterial species *Rhodococcus opacus*, which has been engineered to produce free fatty acids, fatty acid ethyl esters, and long-chain hydrocarbons (Kim et al., 2019). After the culture conditions were optimized to produce 82.9 g/L of triacylglycerols from glucose, an engineered strain was developed to produce 50.2 g/L of free fatty acids from glucose. While this strain has not yet been specifically engineered for fatty alcohol production it clearly has potential for high yields. In some cases, the full potential of these strains remains untapped owing to their resistance to transformation and genome engineering coupled with limited genetic tools for manipulating those genomes. It is likely that combining the appropriate selection processes with transformation and genome-editing technologies will allow us to harness the potential of oleaginous bacteria and yeasts. It is worth noting that a relatively small subset of microbial strains have been tested for the potential to produce fatty alcohols. Microbial strains that naturally produce and secrete glycolipid biosurfactants or other glycolipids have yet to be explored. High throughput screening of existing culture collections may identify strains with improved properties for fatty alcohol production.

The application of synthetic biology and metabolic engineering has enabled us to produce microbial strains capable of catalyzing the conversion of simple carbon feedstock into valuable fatty alcohols and other oleochemicals. Extensive progress in this endeavor over the last decade has been driven by rapid advances in genetic engineering tools coupled with expanding DNA sequence databases from an increasing number of organisms. Harnessing the full potential of microbial cell factories for oleochemical production and development of commercially viable processes will require substantial efforts to improve metabolic pathways toward the desired products, improved catalytic activity of the FAR and CAR enzymes, improved production of more diverse fatty alcohol products, improved feedstock assimilation and improved tolerance to inhibitors and the fatty alcohol products.

## Author Contributions

AK and BM conceived the manuscript. AK, BM, and DS contributed to writing and editing of the original and revised versions of the manuscript and preparation of the figures. All authors contributed to the article and approved the submitted version.

## Conflict of Interest

The authors declare that the research was conducted in the absence of any commercial or financial relationships that could be construed as a potential conflict of interest.
